# Visualization of RNA virus infection in a marine protist with a universal biomarker

**DOI:** 10.1038/s41598-023-31507-w

**Published:** 2023-04-10

**Authors:** Samantha R. Coy, Budi Utama, James W. Spurlin, Julia G. Kim, Harshavardhan Deshmukh, Peter Lwigale, Keizo Nagasaki, Adrienne M. S. Correa

**Affiliations:** 1 Department of Biosciences, Rice University, Houston, TX USA; 2 Department of Oceanography, Texas A&M University, College Station, TX USA; 3 Shared Equipment Authority, Rice University, Houston, TX USA; 4 Faculty of Science and Technology, Kochi University, Nankoku, Kochi 783-8502 Japan

**Keywords:** Water microbiology, Virus-host interactions, Viral tracing

## Abstract

Half of the marine virosphere is hypothesized to be RNA viruses (kingdom *Orthornavirae*) that infect abundant micro-eukaryotic hosts (e.g. protists). To test this, quantitative approaches that broadly track infections in situ are needed. Here, we describe a technique—dsRNA-Immunofluorescence (dsRIF)—that uses a double-stranded RNA (dsRNA) targeting monoclonal antibody to assess host infection status based on the presence of dsRNA, a replicative intermediate of all *Orthornavirae* infections. We show that the dinoflagellate *Heterocapsa circularisquama* produces dsRIF signal ~ 1000 times above background autofluorescence when infected by the + ssRNA virus HcRNAV. dsRNA-positive virocells were detected across > 50% of the 48-h infection cycle and accumulated to represent at least 63% of the population. Photosynthetic and chromosomal integrity remained intact during peak replication, indicating HcRNAV infection does not interrupt these processes. This work validates the use of dsRIF on marine RNA viruses and their hosts, setting the stage for quantitative environmental applications that will accelerate understanding of virus-driven ecosystem impacts.

## Introduction

Viruses have different genomic chemistries (DNA or RNA), architectures (single or double-stranded in circular, linear, or segmented configurations), and vastly different sizes (1.7 kb–2.47 Mbp^1,2^), making it unsurprising that they lack a single genetic signature allowing their universal detection (e.g., ribosomal RNA for cellular life). In marine ecosystems, most efforts to characterize viral impacts have focused on DNA viruses, particularly highly abundant bacteriophages^3^ whose particles can be easily detected.^4,5^ RNA viruses (kingdom *Orthornavirae*, Baltimore classes III, IV, and V^6^), which are hypothesized to primarily infect eukaryotes^7–9^, have been comparatively difficult to enumerate because their smaller size is below the threshold of detection for many quantitative methodologies (i.e. epifluorescent microscopy, flow cytometry^4,10^). However, recent batch nucleic acid measurements from virus-size fractionated seawater suggest RNA viruses comprise half of the marine virosphere^9^.

A vast diversity of marine orthornavirans has been documented using high-throughput sequencing approaches^7,8,11–16^. Bioinformatic tools predict the vast majority of these viruses infect eukaryotes^8^, and given single-celled protists account for a third of marine biomass^17^, it is highly important to determine how frequent these infections are at any given time. Single-cell sequencing represents an important advance that can track viral-host interactions in situ^18–20^; yet, it can be difficult to distinguish viral infection from host association (e.g., consumption or attachment^21^) with this technique. A high throughput approach that can visualize infection within micro-eukaryotic, single-celled hosts can accelerate our understanding of the roles RNA viruses play in shaping marine ecosystems. Development of such a screening-based method relies on the ability to specifically discriminate virus-infected cells.

During viral infection, cells exist as an amalgamation of both cellular and viral processes that constitute a distinct subtype of cell known as a ‘virocell’^22^. Virocells can be distinguished from normal cells by several molecular patterns. For example, transcripts from viral genomes can be visualized inside infected hosts with fluorescent hybridization probes^23–26^. Lipids^27,28^ and elevated reactive oxygen species^29^ can also signify infection. Although these biomarkers have proven useful for quantifying infection in specific, validated systems, they cannot be broadly applied to visually detect environmental virocells because they are either too specific to certain virus-host systems (i.e. transcripts) or indicative of general cell stress (e.g. ROS generation). An infection-specific biomarker that is shared across orthornaviran lineages is needed to broadly estimate the distribution and impact of these viruses in the environment.

RNA dependent RNA polymerase (RdRp) is a gene shared by all orthornavirans and thus has potential as a molecular marker for RNA-virus infected virocells. Yet, although viral RdRp proteins have a deeply conserved polymerase function, their genes exhibit vast sequence divergence^30^, limiting gene-based detections to select lineages at a time^31–33^. Nevertheless, RdRp generates a biomarker for RNA virus infection that is universally conserved: long, double-stranded RNA (dsRNA). Eukaryotic organisms do not produce this molecule and have instead evolved anti-viral immune pathways that are triggered by the presence of dsRNA^34^. The virus-specific association with dsRNA has been known since the late 1960’s^35^, leading to its development as an applied biomarker using antibody-based tools^36^. Animal virologists have widely used dsRNA-targeting antibodies to show that all types of RNA virus infections, and even some DNA virus infections, produce detectable levels of dsRNA^37,38^. Despite its potential as a quantitative tool to assay marine virus infection, this approach has not been applied in aquatic microbial ecology.

Here, we demonstrate how dsRNA-targeting antibodies can provide insight into marine RNA virus infection at the resolution of single host cells using a model protist-RNA virus system: the free-living dinoflagellate, *Heterocapsa circularisquama*, and its positive-sense, single-stranded RNA (+ ssRNA) virus, Heterocapsa circularisquama RNA virus (HcRNAV)^39^. By visually quantifying intracellular dsRNA, we demonstrate that HcRNAV-virocells produced signal over 1000 times the background autofluorescence of non-infected cells. This allowed us to determine that dsRNA is detectable as a biomarker for at least 50% of the infection cycle, and at least 63% of an HcRNAV-inoculated culture forms a virocell sub-population. This work sets the stage for the application of dsRNA-targeting antibodies as a robust, universal tool for estimating and characterizing RNA virus infection in marine and freshwater microeukaryotes.

## Results

### Using general culture dynamics to predict HcRNAV infection prevalence

**Figure 1:**
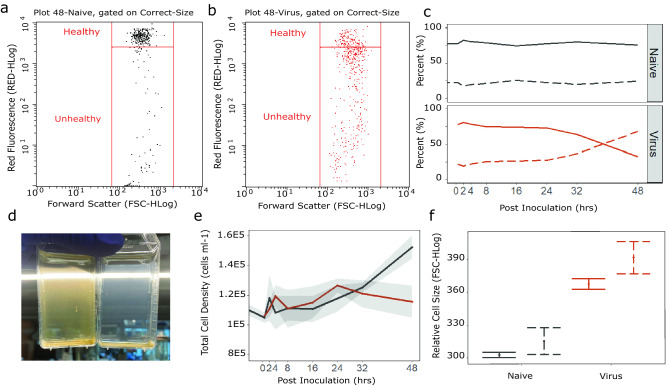
Culture dynamics of *Heterocapsa circularisquama* cells inoculated with HcRNAV strain A-2. Cultures include two sub- populations: a healthy population characterized by high red-autofluorescence, and cells declining in health as indicated by decreased red-autofluorescence. Flow cytometry dot plots depict naive (**a**) and viral-exposed (**b**) cultures containing different proportions of healthy and unhealthy sub-populations at 48 h post-inoculation. (**c**) Cultures are normally dominated by healthy cell populations (solid line) but shift to being dominated by the unhealthy population (dashed line) over the course of viral-infection. (**d**) Side-by-side comparison of naïve (left) and viral-exposed (right) cultures 48 h post-inoculation indicated clearing of cultures, presumably due to a loss of motility. (**e**) Despite the shift from healthy to unhealthy cell populations, there is no decrease in the overall number of viral-exposed cells (red) that would signify viral-mediated lysis. Black line = naïve cultures, shaded area = 95% confidence intervals. (**f**) After 48 h, inoculation treatments exhibit significantly different cell sizes (i.e., forward scattering), indicating viral-exposed cultures are likely on the verge of lysing (p ≤ 0.001, n = 3). Both the healthy (solid error bars) and unhealthy (dashed error bars) sub-populations are larger in viral-exposed cultures. Dot = Average, Error bars = 95% confidence intervals.

*Heterocapsa circularisquama* is a thecate dinoflagellate that is lysed ~ 48 h after infection by HcRNAV^39^. A common infection symptom preceding lysis is a decline in photosystem health, indicated by a decrease in red autofluorescence (e.g., chlorophyll-a). In this experiment, naïve, actively growing algal culture always contained photosynthetically unhealthy and healthy populations, with the latter being the dominant type (Fig. 1a). However, inoculation of cultures with a high virus titer caused a dramatic shift, resulting in photosynthetically unhealthy cells becoming dominant (Fig. 1b). This shift started between 24 and 32 h after HcRNAV exposure, and gradually accumulated to a non-healthy population that comprised 67.9% of the culture (Fig. 1c). In comparison, background levels of unhealthy cells never amounted to more than ~ 25% of the naïve, control culture. The ~ 42% increase in this sub-population (2.66-fold) within viral-exposed cultures was assumed to stem from initially healthy cells that became HcRNAV-infected virocells. Support for this prediction included culture clearing in viral-treated cultures (Fig. 1d), which contrasted with control cultures that remained naturally suspended due to flagellae-mediated motility of *Heterocapsa*. The viral-treated cultures did not lyse over the course of the experiment since the total cell count remained unchanged (Fig. 1e), indicating clearing resulted from a loss of motility and chlorophyll-a degradation. Nevertheless, viral-exposed cultures contained cells whose sizes were 21.3 to 24.2% larger than naïve cells by the end of the experiment (Fig. 1f) and were thus assumed to be on the verge of lysing. Cell swelling significantly occurred in both types of cells (i.e., three replicate comparisons between healthy populations, p = 2.63E−5; unhealthy populations, p = 0.001)*,* and suggested HcRNAV-exposure influenced *Heterocapsa* in dynamic ways. Altogether, these observations allowed us to predict that at least the 42% of the culture that shifted from healthy to a non-healthy state were representative of HcRNAV-infected virocells, though some proportion of healthy cells may also have been infected.

### Super-resolution visualization of dsRNA-targeting antibodies within HcRNAV-infected virocells

**Figure 2:**
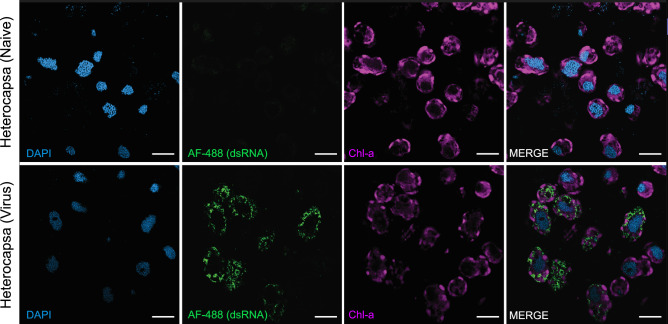
Airyscan, super-resolution imaging 16 h post-inoculation in 0.5 µm sections of *Heterocapsa circularisquama* naïve (top row) and HcRNAV-exposed (bottom row) samples. From left to right, columns indicate DAPI staining, AlexaFluor488 denoting dsRNA binding, chlorophyll-a autofluorescence, and merged images. Scale bars = 10 µm.

All positive-sense RNA viruses progress through three distinct phases of their infection cycle: translation of the viral genome, RdRp-mediated replication of the genome, and finally, packaging of the viral genome into nascently formed virions. The replicative phase should be associated with the presence of dsRNA, yet, RdRp activity has not previously been characterized during HcRNAV infection. Nevertheless, virus-like particles (VLPs) have been shown to accumulate within cells between 12- and 24-h post-inoculation^39^, and because virion loading is a final step, we predicted genome replication would accompany VLP production. Airyscan super-resolution microscopy imaging of cells collected 16 h after HcRNAV exposure confirmed that dsRNA signal (green fluorescence) had accumulated throughout the cytoplasm in 8 of 15 cells (Fig. 2). This contrasted with a lack of discernable green fluorescence in non-infected cultures imaged under identical conditions (0 of 13 cells). Moreover, dsRNA signal was specific to dsRNA, given secondary-only, antibody-stained samples demonstrated a lack of non-specific staining (Supplemental Fig. 1).

In addition to visualizing dsRNA, these Airyscan super-resolution microscopy images enabled qualitative assessment of how dsRNA production correlated with other physiological processes. The dinokaryon structure (i.e., dinoflagellate nucleus containing fibrillar, condensed chromosomes) was indiscernible between treatments; however, DAPI staining intensity was noticeably lower in infected cultures, regardless of whether dsRNA was visually apparent in cells or not (Fig. 2). The HcRNAV-exposed culture also exhibited less chlorophyll-a autofluorescence (Fig. 2), though this contradicted flow cytometric measurements which did not detect a significant difference between treatments at 16 h post-inoculation (Fig. 1c,f). Airyscan super-resolution microscopy may therefore offer higher sensitivity in detecting changes in chlorophyll-a autofluorescence; further analyses are necessary to confirm (or deny) this.

### Quantitative characteristics of dsRNA within HcRNAV-infected virocells

**Figure 3:**
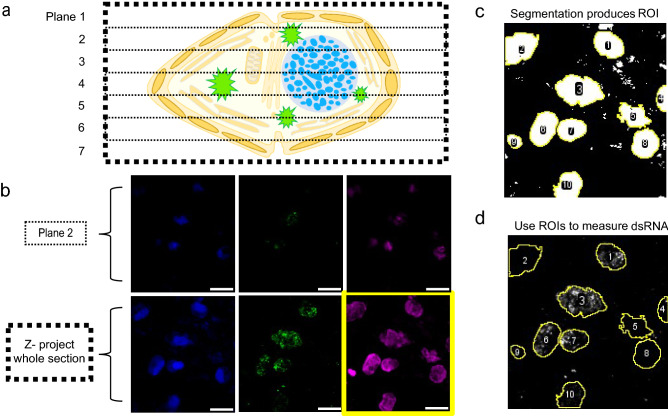
Schematic describing batch processing for quantification of dsRNA. (**a**) Dinoflagellate cells are imaged at z-depth intervals of 1 µm and are denoted here as planes. In reality, whole sections are closer to 20 µm but are reduced here for clarity. (**b**) Imaging of a single plane (e.g., Plane 2) captures only part of the dsRNA signal, while interval imaging of all planes combined with compression into one 2D, maximum intensity Z-projected image allows a complete estimation of dsRNA occurring in the cell. The yellow-outlined chlorophyll-a autofluorescence signal is also required to support automated, quantitative processing. (**c**) Cell boundaries are outlined via segmentation of 2D max intensity Z-projected autofluorescence and saved as regions of interest (ROI). (**d**) ROI are overlaid over each channel image and fluorescence intensity (e.g., dsRNA denoted by AlexaFluor488) is quantified. Scale bars in B = 10 µm.

**Figure 4:**
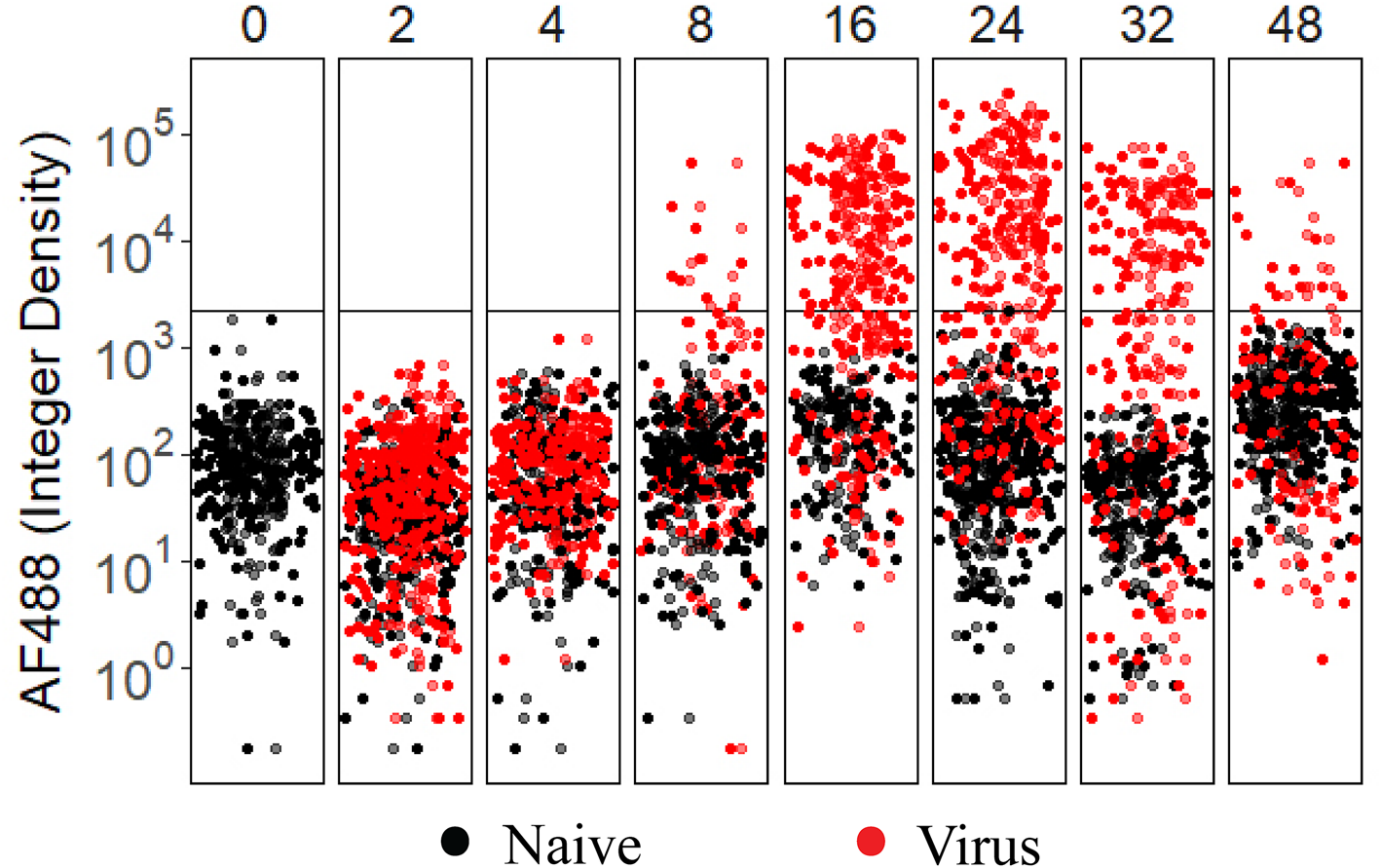
Relative dsRNA signal on a single-cell, normalized scale for naive (black) and HcRNAV-exposed (red) *H. circularisquama* cells sampled across time. Horizontal line indicates the highest background signal for naive cultures and serves as a conservative threshold for categorizing infection state. Variation in color intensity represents an artifact of the position_jitterdodge function of ggplot2 to improve visibility of closely clustered datapoints within each timepoint. (n = 72 to 226 cell observations per timepoint per treatment; 2278 total observations).

In this study, Airyscan super-resolution imaging was used as a proof-of-application, demonstrating that dsRNA-targeting antibodies successfully co-localize, and are specific to, intracellular dsRNA. However, due to logistical constraints, this approach was conducted on a single 0.5 µm thin section, representing a small fraction of the whole *Heterocapsa* cell (i*.e.,* 15-20 µm in diameter). To comprehensively quantify dsRNA presence within a cell, interval imaging must be performed across its entirety. Thus, to quantitatively validate dsRNA-targeting antibodies as a screening tool for orthornaviran infection of marine protists, we leveraged standard confocal microscopy to permit larger surveys (n = approx. 100–200 cells per time point, per treatment) of whole-sections (Fig. 3a–d). Using this approach, the HcRNAV-exposed culture produced a quantifiably distinct sub-population of cells emitting high, intracellular green fluorescence, relative to control cells that were characterized by consistently lower background autofluorescence (Fig. 4). A threshold was conservatively set for positive virocell identification based on the maximum observed green background autofluorescence produced by cells not exposed to HcRNAV. After analytical gating of these populations, virocells were found to produce a ~ 100 to 1000-fold increase in dsRNA signal compared to presumably naïve cells (Fig. 4; Table 1). Comparatively, the exposed, apparently non-infected population was only 1.0–36.1-fold elevated above controls. This suggests variability is present in green autofluorescence and/or the initial stages of dsRNA production is difficult to detect.

**Table 1:** Single cell measurements of dsRNA signal denoted by AF488 by each cell population.

Cell population	AF488	8 h	16 h	24 h	32 h	48 h
Naive: dsRNA (−)	N	156	94	221	157	194
	Average	1.17E+2	2.15E+2	1.54E+2	6.05E+1	3.59E+2
	Std. deviation	1.21E+2	1.81E+2	2.28E+2	52.5E+1	3.16E+2
	Upper limit	7.94E+2	9.15E+2	2.18E+3^†^	2.64E+2	1.55E+3
Exposed: dsRNA (−)	N	82	61	60	62	60
	Proportion (%)	89.9	37.2	36.6	46.9	80.6
	Average	2.74E+2	6.30E+2	4.70E+2	3.72E+2	3.72E+2
	Std. deviation	4.60E+2	6.75E+2	5.44E+2	5.54E+2	4.51E+2
	Upper limit	2.18E+3	2.18E+3	2.18E+3	2.18E+3	2.18E+3
	Fold change*	2.34–18.7	2.93–10.2	3.05–14	6.15–36.1	1.04–6.08
Exposed: dsRNA (+)	N	9	103	104	68	14
	Proportion (%)	10.1	62.8	63.4	53.1	19.4
	Average	1.31E+4	2.68E+4	4.49E+4	2.20E+4	1.34E+4
	Std. deviation	1.70E+4	2.41E+4	4.82E+4	1.72E+4	1.54E+4
	Upper limit	5.54E+4	9.98E+4	2.49E+5	7.64E+4	5.30E+4
	Fold change**	70–474	109–464	114–1617	289–1263	34–148

^†^Maximum single-cell measurement of AF488 in naïve population used to define the exposed dsRNA (+) population.

*Signal difference between the exposed dsRNA (−) and Naive populations based on both the average and upper limit values.

**Signal difference between the exposed dsRNA (+) and Naive population based on both the average and upper limit values.

### Temporal presence of dsRNA within HcRNAV-infected virocells

Virocells appeared at 8 h, peaked at 16–24 h, and declined over the second half of the infection cycle (Fig. 4). Using 48 h as an end point for infection (despite lysis not occurring), 83.3% (from 8 to 48 h) of the infection cycle produced detectable levels of dsRNA. That said, it is possible that infection was not completely synchronized given that we did not remove free viruses shortly after the experiment started. Regardless, we assumed that virus-contact did occur with all available host cells at the same time. This allowed us to infer that the slight decline in positive virocells at 32 h (Fig. 4) could be reflective of a transition from prioritization of viral genome replication to capsid packaging. This transition could therefore be used as a conservative end-point of dsRNA production, but still demonstrated that at least 50% of the infection cycle (from 8 to 32 h) was associated with positive dsRNA signal. Altogether, this suggests that there is a large window of time in which HcRNAV-infected virocells can be reliably detected with dsRNA-targeting antibodies.

### Physiological descriptions of HcRNAV-infected virocells indicated by dsRNA

**Figure 5:**
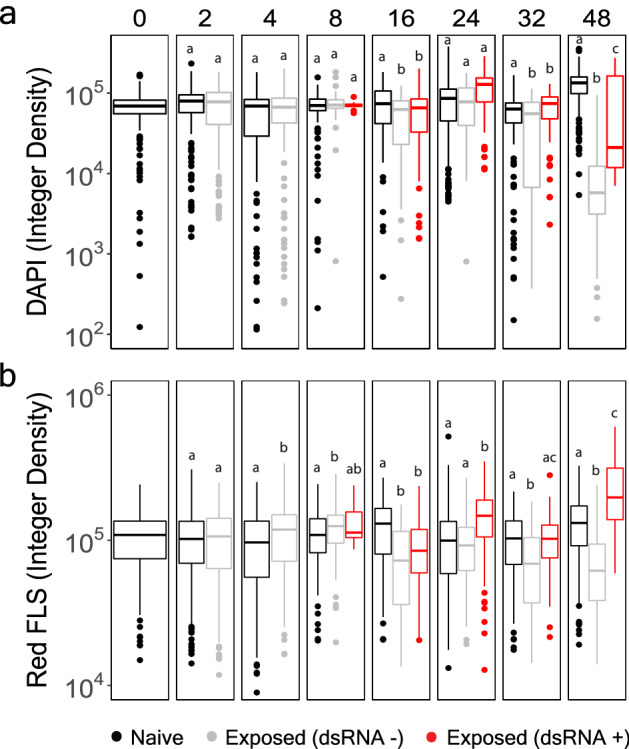
Assessment of chromosomal integrity (**a**) and photosynthetic fluorescence (**b**) between different *Heterocapsa* populations defined by treatment and dsRNA status as interpreted from Fig. 4 data (described in Table 1). Naive cultures are represented in black. HcRNAV-exposed cells that are not associated with dsRNA production are grey, while HcRNAV-exposed cells that significantly produce dsRNA are red. The integer density represents cells normalized by size to account for different cross-sectional planes that might be represented in thin sections. Lower-case letters above each box plot indicate significance groups within each facet, and were determined using Welch’s two-tailed t-test (p-value < 0.05). The data are displayed using the geom_boxplot() function in ggplot2 under the default settings for the box to denote data distribution quartiles, whiskers, and outliers.

Having validated the use of dsRNA-targeting antibodies as a biomarker for aquatic RNA virus infection, these tools were then used to characterize our model system. Based on dsRNA thresholding, nearly two-thirds of the HcRNAV-exposed culture could be categorized as virocells at 24 h post-inoculation (Fig. 4; Table 1). This proportion was 1.5 × greater than our prediction based on the rise of an ‘unhealthy’ cell population detected by flow cytometry (Fig. 1, 42%), suggesting some photosynthetically healthy cells were also infected. To reassess cell health status as a function of viral infection, we partitioned the viral-exposed culture into two sub-populations based on the presence of dsRNA (Fig. 5a,b). The dsRNA-affiliated virocells *(i.e.,* Exposed-Virocells, red in Fig. 5a,b) and non-dsRNA producing cells (*i.e.*, Exposed-negative, grey in Fig. 5a,b) were then compared between one another and with the control (*i.e.*, naïve cells) to detect physiological differences previously observed in flow cytometry and super-resolution microscopy (Figs. 1c, 2). As expected, few changes were observed across cell health metrics during the first 8 h of infection. At 16 h, viral-exposed cells were associated with significantly lower DAPI and chlorophyll signal compared to controls. This trend occurred independent of dsRNA production and thus provided statistical support for these previous observations in super-resolution images (Fig. 2). Over the latter half of the infection cycle, virocells continued to yield cell health metrics that were significantly different from naïve cells. At times virocell health exceeded that of naïve and non-dsRNA affiliated cells (Fig. 5a,b). While unexpected, this trend appears to be most relevant for the 24-h infection point where photosystem health is particularly elevated in virocells. It is possible this may be an artifact of sample processing or microscopy, considering flow cytometry results did not yield any discernable differences in chlorophyll-a between treatment at 24 h (solid black line vs solid red line at 24 h in Fig. 1c). In any case, it is interesting that acute viral replication occurs in apparently healthy cells. By 32 h, the population most reduced in photosynthetic health was the HcRNAV-exposed cells not associated with dsRNA production. The final time point of the HcRNAV-exposed population is highly reduced in both quantifiable DNA and chlorophyll-a content (Fig. 5a,b). Microscopy images at this time point are not only devoid of dinokaryons, but include atypical cell shapes (Supplemental Fig. 2), indicating histological processing likely mediated lysis in already compromised, viral-infected cells.

## Discussion

This study demonstrates the utility of dsRIF in detecting, visualizing, and quantifying active RNA virus infection in marine micro-eukaryotes. The current body of dsRNA literature is dominated by animal host-virus systems; in such systems, a virus species can be tracked by targeting highly specific molecular features (e.g., viral proteins or transcripts). In contrast, a drop of seawater can contain thousands of different microbial eukaryotes^40,41^ and their respective RNA viruses, which has previously stymied efforts to detect and quantify total RNA viruses in environmental systems. The development of dsRIF, a high-throughput universal tool for detecting orthornaviran infections using dsRNA-targeting antibodies, will improve our understanding of many RNA virus-driven processes, ranging from modeling of plankton bloom dynamics (i.e. termination)^42^, to oceanic and atmospheric chemical cycling^43^, to marine metazoan health and disease^12,44^.

*Heterocapsa circularisquama* and HcRNAV constitutes an ideal aquatic host-virus system to validate dsRIF for several reasons. First, HcRNAV is one of only a dozen marine RNA viruses that are isolated in culture^45–47^. Second, the marine RNA virosphere is thought to be dominated by + ssRNA viruses^13,31^; in this way, HcRNAV is representative of a large proportion of the viral community. Finally, HcRNAV is known to acutely infect its host, resulting in production of up to 10,000 infectious progeny within a single cell in only 48–72 h^39^. Based on this magnitude of productivity, we hypothesized that HcRNAV genome replication would be a dominant molecular feature of HcRNAV-virocells, and that dsRNA replicative intermediates would be easily detected. True to our prediction, we demonstrated dsRNA signal was 100–1000 times elevated above the fluorescence background of non-infected cells, and > 50% of the HcRNAV infection cycle was associated with positive signal (Fig. 4). Altogether, this further establishes HcRNAV as a model marine RNA virus, and simultaneously sets the stage for subsequent environmental surveys that can highly resolve how HcRNAV constrains its host during red-tide blooms.

Dinoflagellates are a major taxonomic group in the ocean^40^ that can live free-living (i.e., planktonically) or as endosymbionts within a variety of marine invertebrates. Since *Heterocapsa circularisquama* and its viruses^39,48^ are the only cultured dinoflagellate virus-host system in culture^45,46^, much of what can be known about dinoflagellate-infecting viruses is based on this model host-virus system. HcRNAV strains have been characterized genetically^49–51^, and in terms of their structural biology^52,53^, their impacts on their host^39,50,54,55^, their environmental diversity and distribution^56–59^, and their propensity to control natural red-tide blooms^51,60–62^. Our work builds on this foundational knowledge with a key advance in characterizing the intracellular dynamics of HcRNAV infections. This constitutes the first characterization of viral genome replication in a marine RNA virus. The dsRIF approach allowed us to define distinct phases of the HcRNAV infection cycle, which are distinguished by when dsRNA production begins and ends (Supplemental Fig. 3). The ‘early phase’ of HcRNAV infection, marked by an absence of dsRNA production, is characterized by viral genome translation (0–7 h). The ‘mid-phase’ of infection, marked by dsRNA production, represents prioritization of viral genome replication and potentially concomitant packaging into viral capsids (8–32 h). Finally, the ‘late phase’ of infection is marked by a decrease in dsRNA production in favor of initiating host cell lysis (32–48 h). The infection dynamics resolved here will inform future cell biology studies including transcriptomics, proteomics, and metabolomics efforts. Such studies will generate novel insights into viral-host interplay at distinct infection phases, which can result in diverse infection outcomes (i.e., reflect varying levels of host resistance to viral infection)^25,63^.

This characterization work opens the door for further exploration of the biology of HcRNAV infections. Promising directions include fine-scale resolution of when viral infection transitions from mid to late phase, and identification of the mechanisms that mediate host lysis. We did not remove free-viruses in our experiment, which may have allowed infections to occur in a phased-like manner. Thus, in a future experiment, an adsorption incubation period (e.g., 15–30 min) could be followed by cell pelleting to reduce free-viruses. Subsequent finer-scale sampling (e.g. every 2 h) over the latter half of the infection cycle should then resolve exactly when and to what magnitude RdRp activity ceases. Alternatively, if *H. circularisquama* and HcRNAV become genetically tractable, reporter labeling can be used to define dsRNA kinetics of single, live-cells over the course of infection^64^. Another exciting avenue to pursue would be to determine whether HcRNAV directly controls cell processes during the early to mid-phase of infection, given that the highest incidence of viral replication (i.e., 24 h) occurred in apparently healthy cells with fully functioning photosystems (Figs. 1c, 5b) and apparently intact chromosomes (Fig. 5a). While some DNA viruses can inhibit or degrade these structures during early phase of infection^65^, many other systems function relatively well up until later stages of infection^66–68^. Altogether, the dsRIF approach presented here advances our understanding of the biology and evolution of dinoflagellates and their viruses, and sets the stage for numerous additional lines of related inquiry.

**Figure 6:**
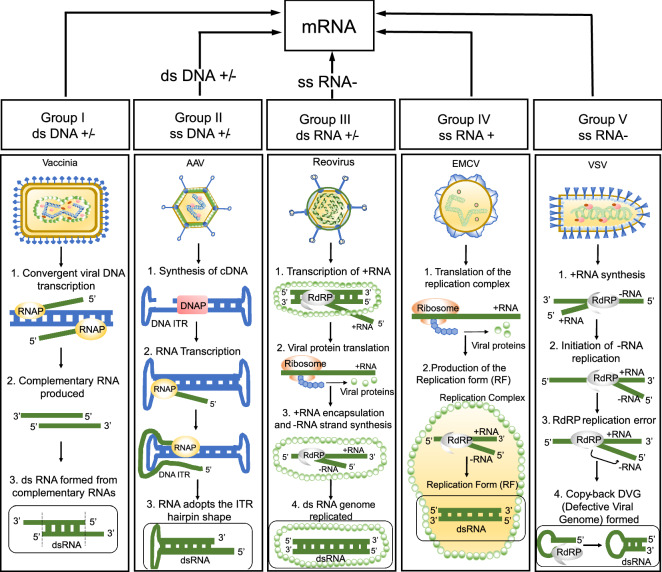
Mechanisms of dsRNA production across representative viruses for each Baltimore classification, excluding retro-viruses. The top part of the diagram depicts the Baltimore-based pathway each viral type uses to produce mRNA, while the bottom shows mechanisms for how each of the representative viruses make dsRNA (circled at the bottom of each panel). DNA is indicated in blue, while RNA is denoted by green nucleic acid strands. *DNAP* DNA polymerase, *RNAP* DNA-dependent-RNA-polymerase, *RdRP* RNA-dependent RNA polymerase. These mechanisms are specific to these viruses but may be shared by other lineages in each group. References for the mechanisms explaining these examples are included: Vaccinia Virus^38^, Adeno-associated Virus (AAV)^38,75^, Reovirus^38^, Encephalomyocarditis Virus (EMCV)^38,69^, and Vesicular Stomatitis Virus (VSV)^69^. The figure was generated in Microsoft Powerpoint Version 2301.

We used a + ssRNA virus as the test case for quantifying dsRNA production in the marine RNA virosphere because of the predominance of this viral group in the ocean. However, dsRIF will likely detect other types of marine RNA virus infections (i.e., those with dsRNA and -ssRNA genomes in Fig. 6), based on demonstrations from animal viruses^37,38^. However, some RNA virus infections, particularly those caused by negative sense, single stranded RNA viruses (-ssRNA), may at times produce false negatives for dsRNA production. The extent to which -ssRNA viruses produce detectable dsRNA was initially thought to be low, because early immunostaining attempts failed to resolve positive signal in most cases^38^. In the few cases where dsRNA was detected, such as in Vesicular Stomatitis Virus (VSV) infections, it was proposed that dsRNA was produced solely from defective interfering particles (DIPs)^69^, which are a common, but not universal, genomic variant of (−)ssRNA viruses. However, when it was subsequently realized that many (−)ssRNA viruses encapsulate nascently formed nucleic acids (e.g., Influenza A Virus), which masks them from antibody binding, an enzymatic pretreatment (i.e., proteinase K) was shown to unmask dsRNA. This suggests the mechanisms for producing dsRNA can be quite diverse across (−)ssRNA viruses and thus may require additional troubleshooting to confirm lack of detection constitutes a biological truth, rather than a false negative^37^. To date, proteinase K pre-treatment combined with the higher sensitivity of the 9D5 anti-dsRNA antibody has confirmed dsRNA production within at least five (-)ssRNA virus families^37,70^; the remaining majority of (-)ssRNA virus families remain untested. It is possible certain lineages of + ssRNA and dsRNA viruses have evolved similar encapsulating strategies, although HcRNAV did not require this pre-treatment. Selective pressure to mask dsRNA may be a function of low productivity infections that fail to neutralize host immunity or higher immune surveillance where dsRNA production is occurring. dsRNA may even sometimes be formed by DNA viruses (i.e., ssDNA and dsDNA), though this is assumed to be accidental, as opposed to being a hallmark of RNA virus infection. In any case, for any viral groups or lineages that do not appear to produce dsRNA signal, further investigations will be necessary to confirm that encapsulation or other masking strategies are not at play. Ensuring that we are able to detect all dsRNA produced by viruses is critical to accurately quantify total orthornaviran infections in aquatic environments, and to disentangle the relative contribution of different viral groups to environmental infection rates.

Viruses are often cited as the most abundant biological entities on Earth^3^, responsible for cycling up to a quarter of the biomass in the sunlit ocean every day^43^. Yet, these measurements are mostly based on prokaryotic viruses; the contributions of eukaryote-infecting viruses remain less defined. If we assume RNA viruses comprise half of the ocean virosphere^9^, then their abundance should be close to 10^7^ RNA virus particles mL^−1^. This is an astounding number, given their putative hosts (e.g., protists, fungi) typically occur at abundances of 10^3^ to 10^4^ cells mL^−1^. However, if a large proportion of this host population is infected, given that HcRNAV infections can produce upwards of 10^4^ viruses per single-cell, the 10^7^ RNA virus particles ml^-1^ estimate is possible^44^. The dsRIF approach constitutes a key advance towards testing this important hypothesis, and promises to play an important role in delineating how RNA viruses shape marine biodiversity and ecosystem functioning.

## Materials and methods

### Infection experiments and sampling

The dinoflagellate *Heterocapsa circularisquama (*HU9433-P) was grown in Daigo IMK (Fujifilm, Japan) amended with 0.2% (v/v) soil extract^71^ in vented, non-treated 25 cm^2^ culture tissue flasks (Corning™, n = 15). Dinoflagellate cultures were maintained at 25 °C in a Percival incubator (Model I40-LL fitted with Philips F25T8/TL841 fluorescent bulbs) under a 12:12 h light: dark cycle initiated at 0700 h. Growth dynamics were estimated with a GUAVA easyCyte HT flow cytometer using forward scatter, side scatter, and 685 nm fluorescence detection. When culture density reached ~ 10^5^ cells mL^−1^, the cultures were transferred into a large Erlenmeyer flask, at which point a 45 mL sample was collected, fixed in paraformaldehyde (Macron™, final conc. 0.5% v/v), and stored at 4 °C as a pre-infection time point. The remaining culture was redistributed across the smaller culture tissue flasks (n = 14) and treated at 1100 h; half of the cultures were inoculated with HcRNAV (strain A-2) at an estimated multiplicity of infection (MOI) of ~ 26 based on most probable number estimates^72^, while the remaining control flasks were diluted with an equal volume of fresh Daigo IMK medium. Cultures were maintained at standard incubation conditions aside from brief sampling intervals at 2, 4, 8, 16, 24, 32, and 48 h post inoculation. At each time point, a 45 mL sample was collected from both a control and virus flask and fixed as described above; all samples were stored in the dark at 4 °C until further processing.

### Sample embedding and thin sectioning

Fixed cells were pelleted using a Sorvall Legend X1R centrifuge mounted with a TX-400 rotor (Thermo Scientific) at 3000×*g* for 5 min. Supernatants were decanted and algal pellets resuspended in 1 mL of 10% neutral buffered formalin (Epredia™ HiPur™). Algal pellets were gross processed and embedded by HistoWiz Inc. using a Standard Operating Procedure and fully automated workflow. Paraffin blocks were sectioned in-house using a Leica microtome at a width of 4 μm followed by adhesion to charged slides (HistoBond®). Slides were stored at room temperature in the dark until further processing.

### Immunostaining

Thin sections were dewaxed with Histosol™ twice for five minutes and rehydrated using a decreasing ethanol gradient (100 to 0%) diluted with RNase-free water. Rehydrated slides were washed 10 min with 1XPBS amended with 0.1% tween (PBST) three times. Next, sections were blocked using PBST amended with 0.1% BSA and 5% Heat Inactivated Goat Serum (MP Biomedicals™). Blocked tissue was immunostained at room temperature for two hours with anti-dsRNA primary monoclonal antibody (Absolute Antibody Ab00458-1.1) at a 1:1000-fold concentration diluted with blocking solution. Non-bound antibody was washed four times with PBST for 15 min each, and re-blocked for 15 min. Next, a 1:200-fold concentration of rabbit anti-mouse secondary antibody conjugated with AlexaFluor488 (AF488) (Thermo A-11059) was added and incubated at 4 °C overnight. The next morning, slides were washed with PBST for 10 min, followed by two rinses with PBS. DAPI stain was applied for 15 min, followed by another 10-min PBST wash and two PBS rinses. Slides were mounted with Fluoromount-G (Southern Biotech) and allowed to cure for at least 24 h. Secondary antibody-only controls were produced for all samples to verify a lack of non-specific reporter binding.

### Microscopy

Sub-cellular localization of dsRNA was visualized in control and viral-inoculated cultures 16 h-post infection with a Zeiss LSM800 inverted confocal microscope (Carl Zeiss) equipped with an Airyscan-1 detector which uses an array detector consisting of 32 hexagonal micro lenses arranged in a circular disk. Airyscan super resolution images were captured using a Plan-Apochromat 63x/1.40 Oil DIC M27 objective in 2028 × 2028 pixel frame resolution at 0.05 µm pixel size following excitation/emission-range at the following wavelengths: ex 405/em 410–480 nm (DAPI), ex 488/em 493–70 nm (AF488), and ex 561/em 576–700 nm (red autofluorescence). Standardized settings were determined for DAPI and red autofluorescence based on uninfected cultures, whereas AlexaFluor 488 intensity was standardized by virus-infected samples. Raw images captured were then processed under Airyscan Processing module available in Zen 2.6—Blue edition (Carl Zeiss) with 2D SR processing option, and the Airyscan filtering (Wiener filter associated with deconvolution) was set to Standard. Raw images captured using Airyscan GaAsP detecter and subsequent deconvolution process resulted in two-fold resolution increase and eight-fold increase of signal-to-noise-ratio relative to conventional confocal microscopes while retaining confocal functionality^73^. The Airyscan processed images were then rendered in a colorblind accommodating color palette in FIJI^74^ and verified with COBLIS (https://www.color-blindness.com/coblis-color-blindness-simulator/). Only one representative image was collected at this time point for each treatment in order to make qualitative comparisons about dsRNA staining.

To quantify dsRNA signal within cells, whole section z-stack imaging was conducted at 1 µm intervals on a Nikon A1-Rsi inverted confocal microscope (Nikon) using a Plan-Apochromat LWD 40x/ NA 1.15 DIC water immersion objective (Nikon). A single track was used to image cells using a pinhole size of 1AU combined with excitation/emission-range: ex 405/em 425–475 nm (DAPI), ex 488/em 500–550 nm (AF488), and ex 560/em 570–620 nm (red autofluorescence) laser-line. Images were captured in 512 × 512 pixel frame resolution at 2.2 µs pixel-dwell with 2 × averaging using GaAsP detectors. Two images were collected per sample, yielding roughly 100–200 imaged cells per timepoint per treatment to support analytical measurements. The potential for false positives in this dataset was low, because segmentation resulted in minimal overlap of dsRNA-producing cells with neighboring, non-infected cells.

### Data and statistical analysis

Z-stack confocal microscopy images were imported into Fiji^74^, where they were batch processed using an automated macro available on Github (https://github.com/samantharosecoy). This automated process involved importing z-stack images, processing them into 2D maximum intensity projection images, segmenting cells by red autofluorescence and co-localizing with each channel to quantify chlorophyll-a fluorescence, DAPI and dsRNA signal. Co-localization data was exported into a .csv file for further statistical analyses in R Studio ver. 4.0.1. Descriptive tests include measures of central tendency, data spread, fold over background, and percent of positive virocells in each sample. Parametric tests of significance were conducted between treatments and time as indicated in the figure legends.

## Supplementary Information

Supplementary Figures.

## Data Availability

Raw z-stack confocal microscopy images and automated macro available on Github (https://github.com/samantharosecoy).
