# Supplementary material for: Visualization of RNA virus infection in a marine protist with a universal biomarker
Source: Sci Rep. 2023 Apr 10;13:5813. doi: 10.1038/s41598-023-31507-w (PMC10086069; doi:10.1038/s41598-023-31507-w)
Supplement: Supplementary file 1 — Supplementary Figures. [file 41598_2023_31507_MOESM1_ESM.pdf]

Supplementary Figures:

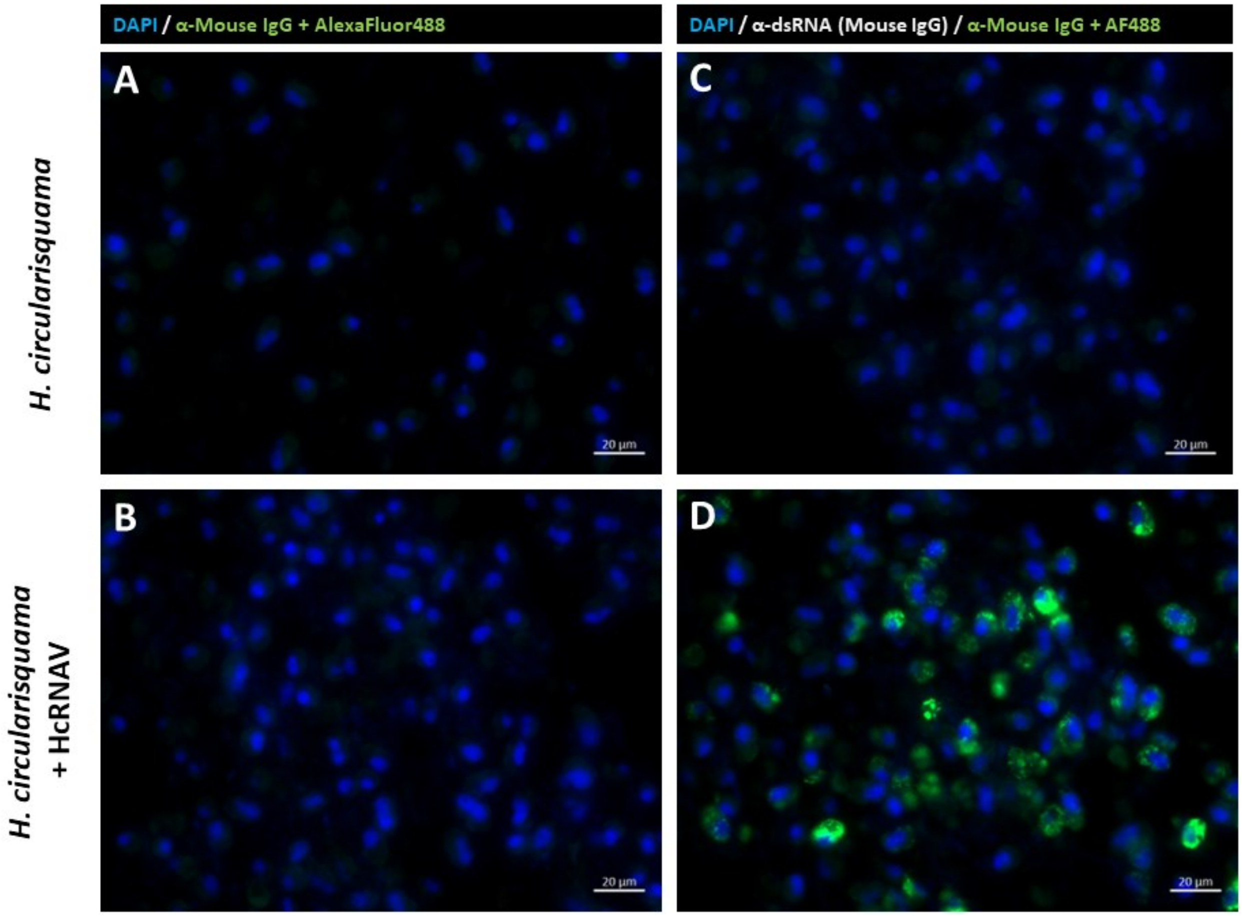

Supplemental Figure 1: Verification of antibody binding specificity to viral dsRNA. Applying just the secondary-reporter antibody results in minimal, non-specific binding in both naive and HcRNAV-exposed cultures because the target antigen, MAb-9D5 antibody, is not present. (A, B). Similarly, MAb-9D5 exhibits little non-specific binding in naive cultures because these cells do not produce the target antigen, dsRNA (C). HcRNAV infection results in production of dsRNA, allowing MAb-9D5 to bind, followed by binding of secondary reporter antibody conjugated with AlexaFluor488 (D). Scale bar = 20 µm.

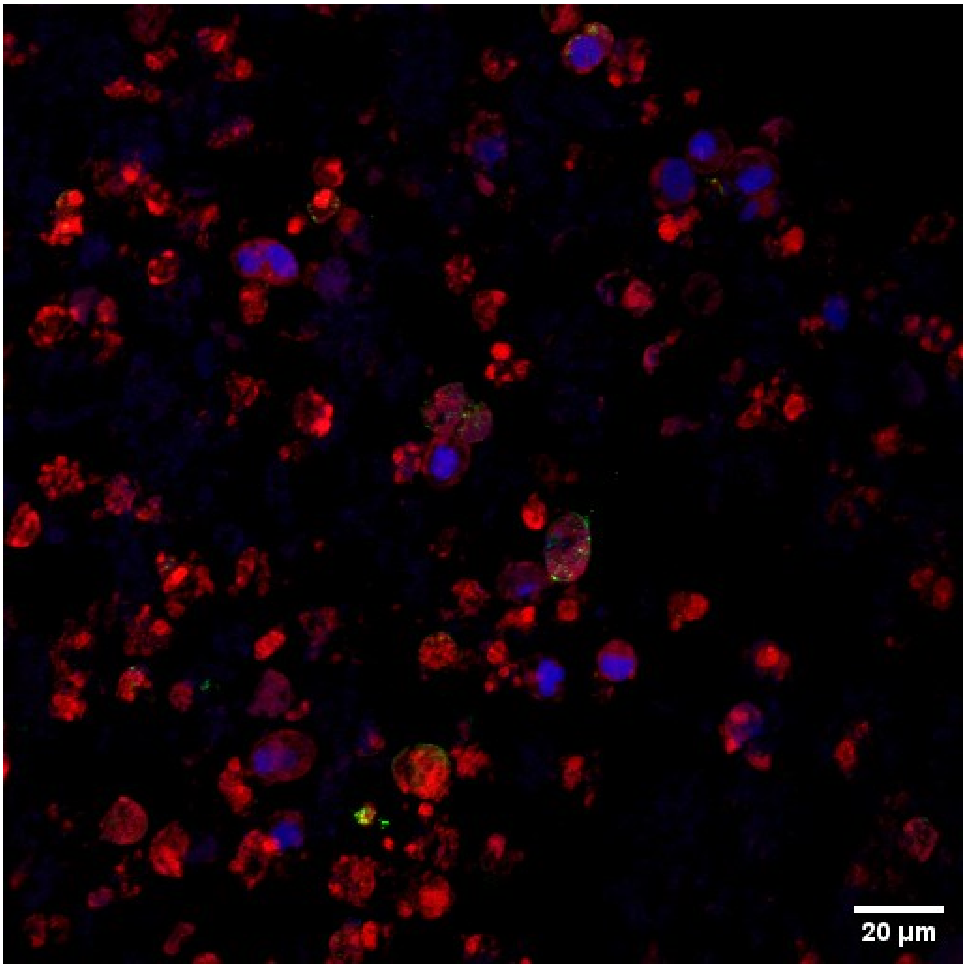

Supplemental Figure 2: Merged 2D maximum intensity projections of HcRNAV-exposed cultures 48 hours after infection showing cells are obviously lysed, given that many lack their dinokaryon and are atypically shaped.

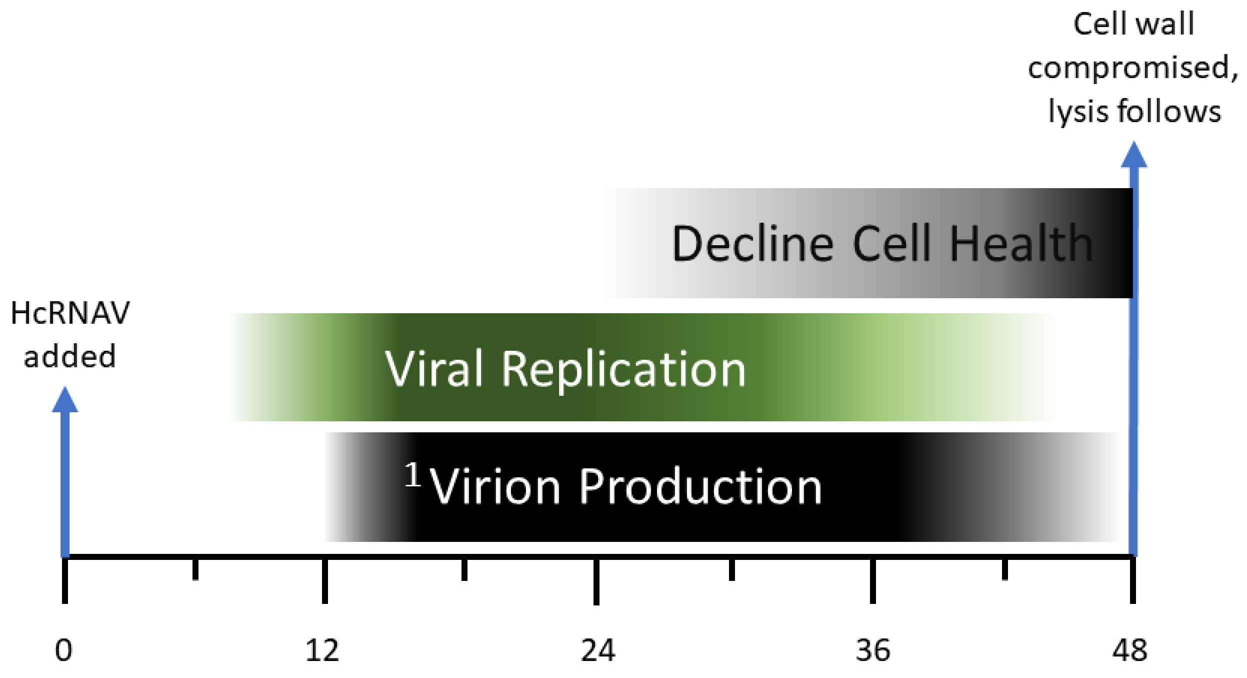

Supplemental Figure 3: Schematic outlining the progression of the HcRNAV infection cycle of *H. circularisquama.* Viral processes are phase colored to represent peak activity with more saturated color. Virion production observations are based on literature reports.
